# Development of an Inflammation-Associated Colorectal Cancer Model and Its Application for Research on Carcinogenesis and Chemoprevention

**DOI:** 10.1155/2012/658786

**Published:** 2012-02-28

**Authors:** Takuji Tanaka

**Affiliations:** ^1^The Tohkai Cytopathology Institute: Cancer Research and Prevention (TCI-CaRP), 5-1-2 Minami-uzura, Gifu City, Gifu 500-8285, Japan; ^2^Department of Oncologic Pathology, Kanazawa Medical University, 1-1 Daigaku, Uchinada, Ishikawa 920-0293, Japan; ^3^Department of Tumor Pathology, Gifu University Garduate School of Medicine, 1-1 Yanagido, Gifu City, Gifu 501-1194, Japan

## Abstract

Chronic inflammation is a well-recognized risk factor for development of human cancer in several tissues, including large bowel. Inflammatory bowel disease, including ulcerative colitis and Crohn's disease, is a longstanding inflammatory disease of intestine with increased risk for colorectal cancer development. Several molecular events involved in chronic inflammatory process may contribute to multistep carcinogenesis of human colorectal cancer in the inflamed colon. They include overproduction of reactive oxygen and nitrogen species, overproduction and upregulation of productions and enzymes of arachidonic acid biosynthesis pathway and cytokines, and intestinal immune system dysfunction. In this paper, I will describe several methods to induce colorectal neoplasm in the inflamed colon. First, I will introduce a protocol of a novel inflammation-associated colon carcinogenesis in mice. In addition, powerful tumor-promotion/progression activity of dextran sodium sulfate in the large bowel of *Apc* ^*Min*/+^ mice will be described. Finally, chemoprevention of inflammation-associated colon carcinogenesis will be mentioned.

## 1. Introduction

Relationship between inflammation and cancer has been suggested for a long time [1]. Since Marshall and Warren [2], who discovered *Helicobacter pylori* and reported its infection closely associated with gastric cancer development, won the Nobel Prize in Physiology or Medicine in 2005, there have been an increasing number of reports on PubMed as to the relationship between inflammation and carcinogenesis in a variety of tissues (Table 1) and it has been featured in major journals. 

**Figure 1:**
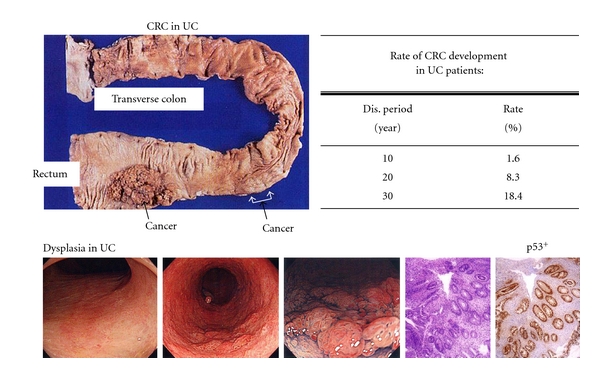
UC patients are high-risk groups of colorectal cancer (CRC) development.

In terms of the large bowel, it has been found that the risk of colorectal cancer increases in relation to the degrees of inflammation and the disease duration (duration/risk = 10 years/1.6%, 20 years/8.3%, and 30 years/18.4%) in inflammatory bowl diseases (IBDs) such as ulcerative colitis (UC) and Crohn's disease (CD) (Figure 1) [3]. I have been interested in inflammation-associated colorectal carcinogenesis for a long time, since even younger patients with UC have high risk of colorectal cancer [4].

**Figure 2:**
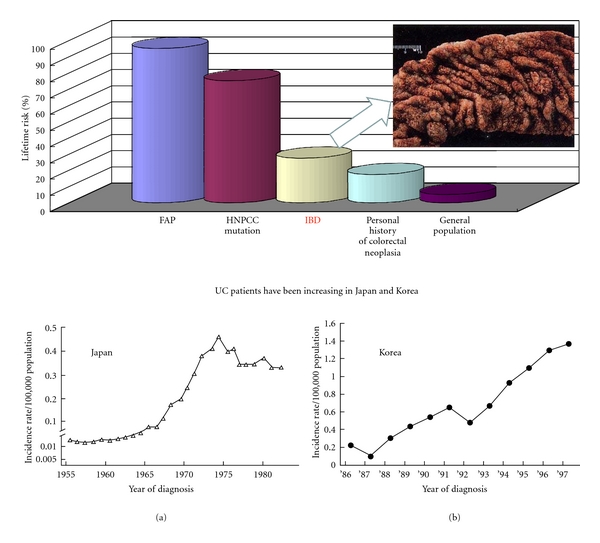
Risk of colorectal cancer.

Patients with UC as well as those with colorectal cancer have been increasing in Asian countries including Japan, similarly to Western countries (Figure 2) [5]. Therefore, it is necessary to investigate the mechanisms of colorectal cancer development with the background of inflammation for establishing the countermeasure strategy such as chemoprevention [6–8]. To this end, a novel animal model is required but there have been few useful animal models. In this paper, I would like to introduce details of my short-term mouse and rat colorectal cancer models with the background of colitis mimicking human UC and our exploration of chemopreventive agents using these models [6–8].

## 2. Process of Human Colorectal Carcinogenesis

**Figure 3:**
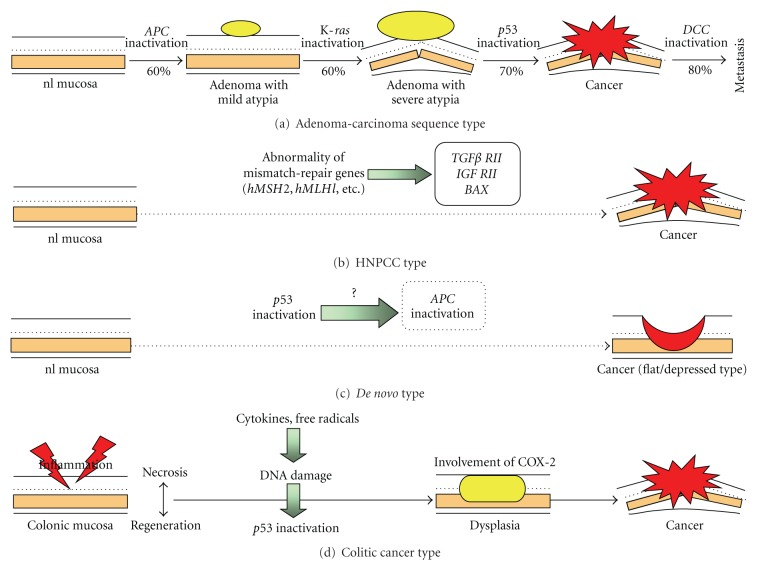
Carcinogenic steps of four types of human colorectal cancer.

There are at least four types of human colorectal carcinogenesis (adenoma-carcinoma sequence type, hereditary nonpolyposis colorectal cancer (HNPCC) type, *de novo* type, and colitic cancer type) (Figure 3) [9]. Of them, the colitic (colitis-associated) cancer type arises from the background of colitis and DNA injury is induced by production of free radicals by the inducible nitric oxide synthase (iNOS) system in the colonic mucosa with persistent inflammation, followed by *p53* mutation and development of dysplasia, a precancerous lesion. Furthermore, dysplasia is advanced by cyclooxygenase- (COX-) 2, iNOS, and several cytokines produced in the infiltrated inflammatory cells and accumulation of genetic abnormality, such as a loss of the *DCC* gene, leads to invasive colorectal cancer. Unlike common colorectal cancer (adenoma-carcinoma sequence type), it has been thought that the *APC  *and K-*ras* genes and microsatellite instability (MSI) are hardly involved in this type, but there remains to be further discussed [9].

## 3. Development of an Inflammation-Associated Colorectal Cancer Model

**Figure 4:**
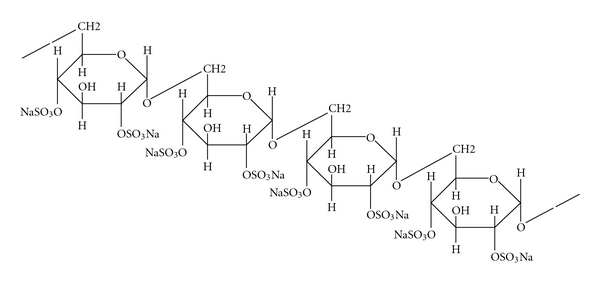
Chemical structure of dextran sulfate sodium (DSS), a sulfated polysaccharide, and its biological activities. DSS (1–5% in drinking water or diet) induces colitis in rodents. Treatment with DSS (1% in diet) after DMH exposure produces colonic adenocarcinoma [44]. The tumorigenicityof DSS is non-genotoxic effects [20]. Cycle treatment with 3% DSS (MW 54,000, 7 days) and distilled water (14 days) produces colonic tumors [45]. DSS increases the number of ACF induced by AOM [19].

Rats have mostly been employed for an animal colorectal carcinogenesis model, and azoxymethane (AOM), methylazoxymethanol (MAM) acetate, and 1,2-dimethylhydrazine (DMH) have been widely used as colorectal carcinogenic substances (Table 2) [10]. About 30 weeks are required for development of colorectal cancer in about half of rats that are initiated with the colonic carcinogens. On the other hand, in experiments and studies using mice, multiple administrations of similar colorectal carcinogens are required and it takes a long term of 40 weeks or longer to develop colorectal cancer [11]. Therefore, I tried to develop a novel mouse model that would develop colorectal cancer in a short term in the inflamed colon [12]. To settle the issue of the influence of peroxisome proliferator-activated receptor (PPAR) agonists on colorectal carcinogenesis, which has been a topic on the journal *Nat Med* since 1998 [13–15], we confirmed that colitis inducing dextran sodium sulfate (DSS), employed in an experiment using rats with aberrant crypt foci (ACF) as a biological marker (Figure 4) [9, 16–18], had tumor promoter activity to accelerate development of ACF and hypothesized that a combination of DSS and AOM would induce colorectal cancer in a short-term period in mice as well [19].

**Figure 5:**
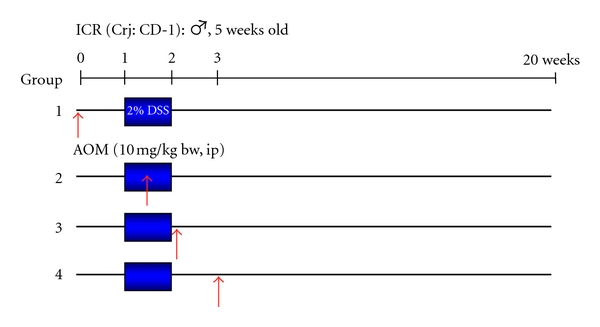
Experimental protocol to develop an inflammation-associated mouse colon carcinogenesis model, to develop a new inflammation-related mouse colon carcinogenesis model [12].

**Figure 6:**
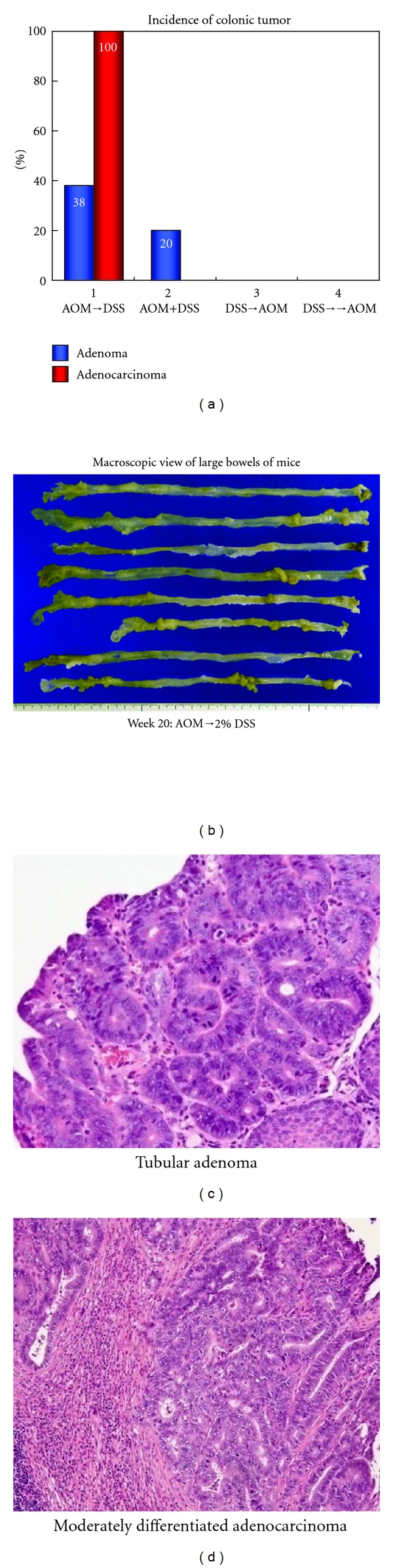
Macroscopic view, incidence, and histopathology of colonic tumors in the groups of mice that received four different treatment schedules of AOM and DSS.

Since DSS is a nongenotoxic carcinogen [20], male ICR mice were divided into three groups that received different administration patterns: DSS→AOM, AOM during DSS administration, and AOM→DSS (Figure 5). In the groups of DSS→AOM and AOM→DSS, there was a one-week interval between the treatments [12]. DSS was given at the concentration of 2% in drinking water (distilled water) for one week and AOM was administered intraperitoneally once at a low dose of 10 mg/kg body weight, which could not induce colorectal tumors, namely, the low-dose initiation. Interestingly, many colorectal tumors (tubular adenomas and tubular adenocarcinomas) developed in the distal colon, where DSS could induce severe colitis, of mice in the group of AOM→DSS. On the other hand, mice of other groups (the DSS→AOM and the AOM during DSS administration groups) did not develop colorectal tumors. The findings confirm potent tumor-promotion activity of DSS (Figure 6). At the same time, the results reconfirmed importance of inflammation in colorectal carcinogenesis [12]. In addition, accumulation of *β*-catenin in the nuclei of colorectal adenocarcinoma cells was observed.

**Figure 7:**
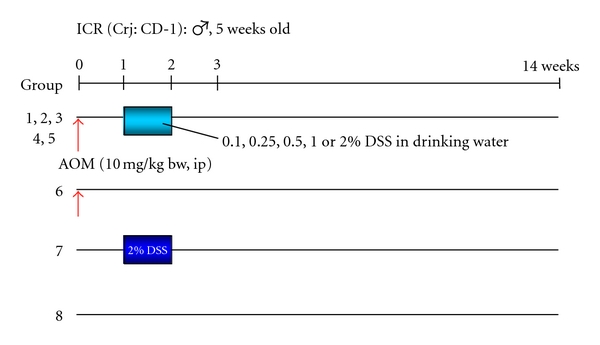
Experimental protocol for determining dose-response of DSS in mice initiated with AOM [21].

**Figure 8:**
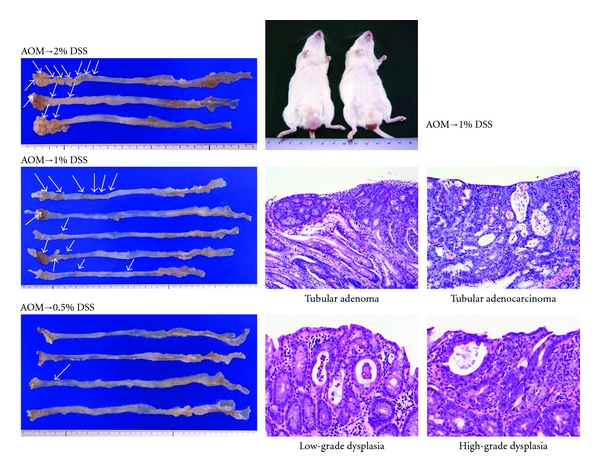
Macroscopic view and histopathology of colonic tumors developed in mice that received AOM and DSS (0.5%, 1%, or 2% DSS in drinking water).

**Figure 9:**
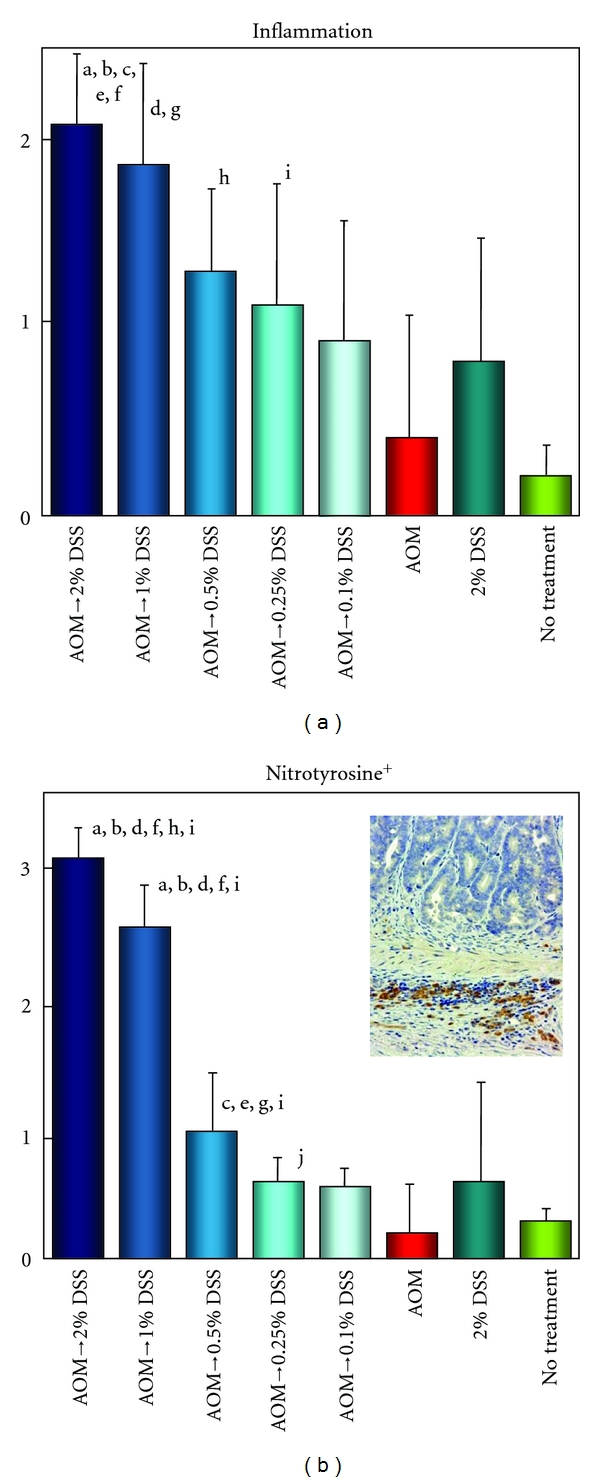
Inflammation and nitrotyrosine-positive scores in the colon of mice that received AOM and/or DSS (0.1%, 0.25%, 0.5%, 1%, or 2% DSS in drinking water). (a) Significantly different: a (*P* < 0.05), versus AOM→0.5% DSS group; b (*P* < 0.05), versus AOM→0.1% DSS group; c (*P* < 0.01) and d (*P* < 0.05), versus AOM alone group; e (*P* < 0.05), versus 2% DSS alone group; and f (*P* < 0.001), g (*P* < 0.005), h (*P* < 0.01), and i (*P* < 0.05), versus no treatment group. (b) Significantly different: a (*P* < 0.001), versus AOM→0.5% DSS group; b (*P* < 0.001) and c (*P* < 0.05), versus AOM→0.25% DSS group; d (*P* < 0.001) and e (*P* < 0.01), versus AOM→0.1% DSS group; f (*P* < 0.001) and g (*P* < 0.05), versus AOM alone group; h (*P* < 0.005), versus 2% DSS alone group; and i (*P* < 0.001) and j (*P* < 0.05), versus no treatment group.

Dose dependence of tumor-promotion activity of DSS after a single intraperitoneal administration of AOM (10 mg/kg body weight) was subsequently examined at five doses (0.1%, 0.25%, 0.5%, 1%, and 2%) of DSS (Figure 7) [21]. The findings indicated that tumor-promotion activity DSS was not observed at the concentration 0.25% or lower and only one tubular adenoma developed in a mouse that received AOM and 0.5% DSS. Colorectal tumors were developed in all mice by the treatment with 1% DSS and 2% DSS after AOM initiation and the number of colorectal adenocarcinoma was much greater in the group of mice treated with 2% DSS (Figure 8). The severity of colonic inflammation was determined by the histological inflammation score and immunohistochemical nitrotyrosine-positive reactivity. Both the inflammation score and nitrotyrosine-positive score in inflammatory cells that infiltrated colonic mucosa were higher in mice that received higher doses of DSS after AOM, suggesting that inflammation and nitrosation were involved in the tumor-promotion activity of DSS (Figure 9).

**Figure 10:**
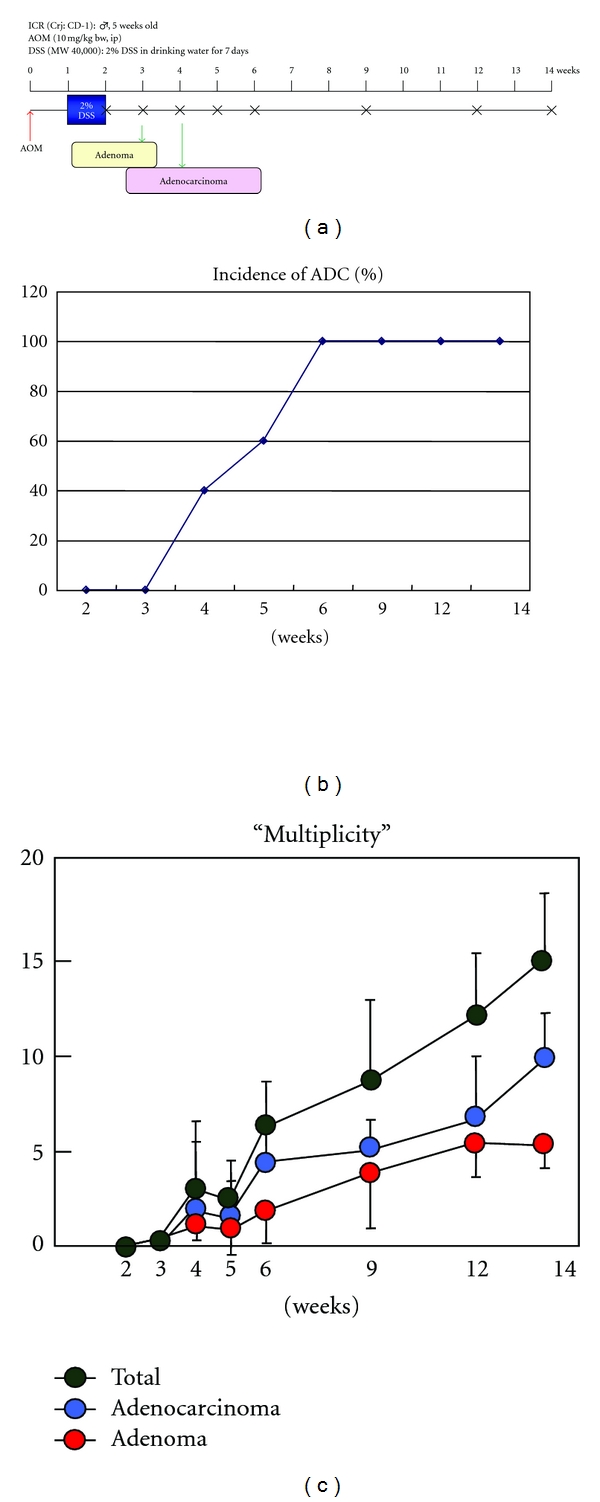
Experimental protocol of time-course observation of AOM/DSS-induce inflammation-associated colorectal carcinogenesis and tumor development (incidence and multiplicity) during the study [11].

**Figure 11:**
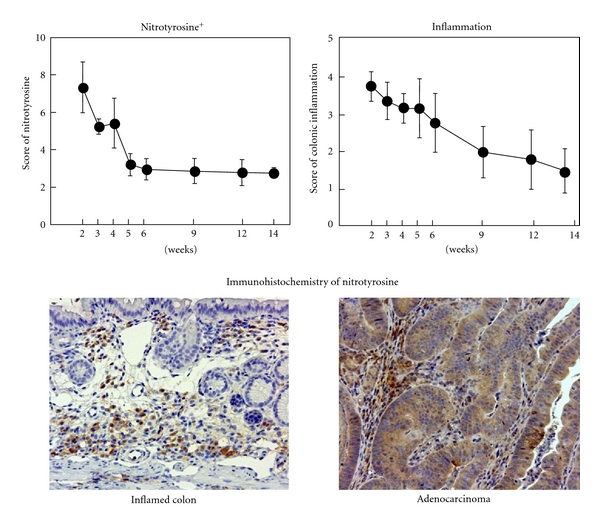
Scores of nitrotyrosine-positivity and inflammation in the inflamed colon and colonic tubular adenocarcinoma.

**Figure 12:**
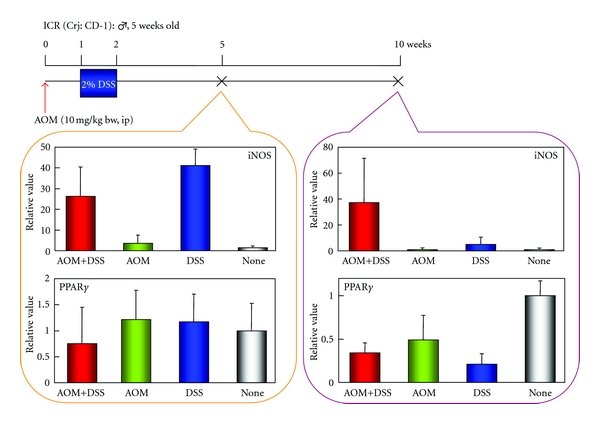
Real-time PCR analysis of iNOS and PPAR*γ* in the colonic mucosa of mice that received AOM and DSS at weeks 6 and 10.

Time-course observation during AOM/DSS-induced mouse colorectal carcinogenesis was conducted to determine when colonic tumors occur in the inflamed colon of mice that received 2% DSS after the AOM initiation [22]. Male ICR mice were initiated with a single intraperitoneal injection of AOM (10 mg/kg body weight) and followed by one week administration with 2% DSS in drinking water. Our time-course observation revealed that colorectal adenoma and adenocarcinoma developed three and four weeks after AOM administration, respectively, and the numbers increased in a time-dependent manner during the follow-up period up to 14 weeks (Figure 10). Interesting finding of this study was that the high inflammation score and high nitrotyrosine-positive score lasted until five to six weeks after the cessation of DSS administration (Figure 11). Since mucosal ulcer caused by DSS administration was microscopically repaired at this point, persistence of the high nitrotyrosine-positive score, rather than the high inflammatory score, is intriguing as well as strong iNOS expression and weak PPAR*γ* expression in the colonic mucosa at five and 10 weeks after the AOM administration (Figure 12). 

**Figure 13:**
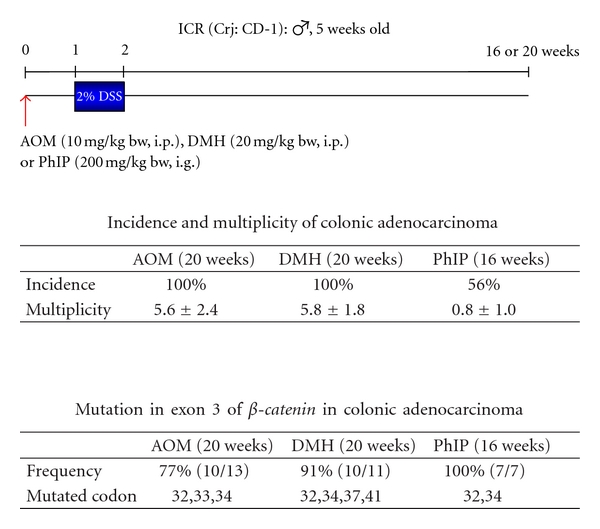
DSS is a powerful promoter in colon carcinogenesis in mice initiated with various colonic carcinogens, azoxymethane (AOM), 1,2-dimethylhydrazine (DMH), and 2-amino-1-methyl-6-phenylimidazo[4,5-*b*]pyridine (PhIP) [12, 21–24, 26].

**Figure 14:**
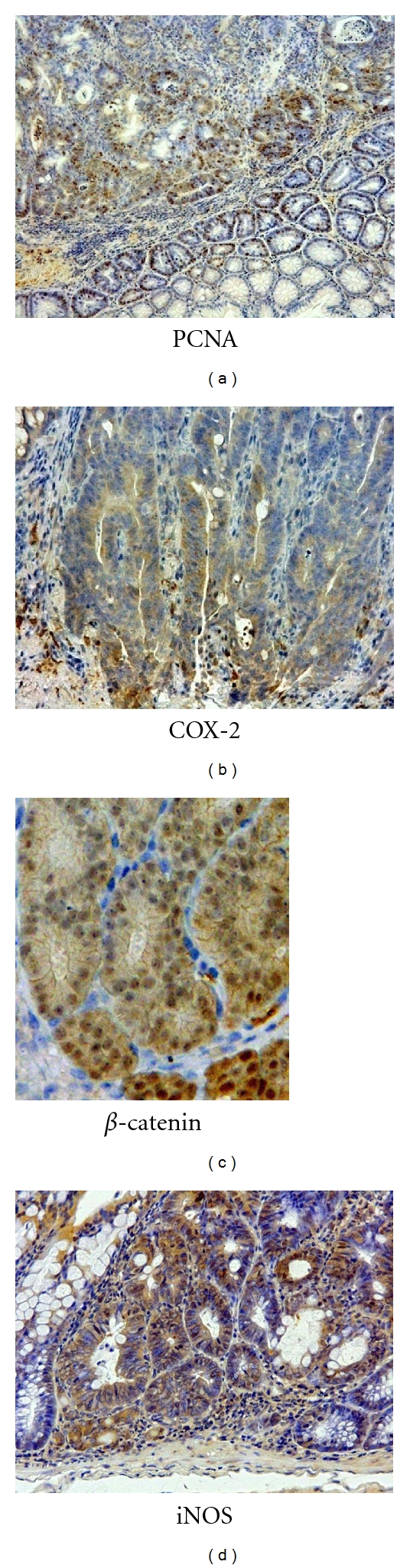
Immunohistochemistry of PCNA, *β*-catenin, COX-2, and iNOS in colonic adenocarcinomas of mice induced by AOM and DSS.

**Figure 15:**
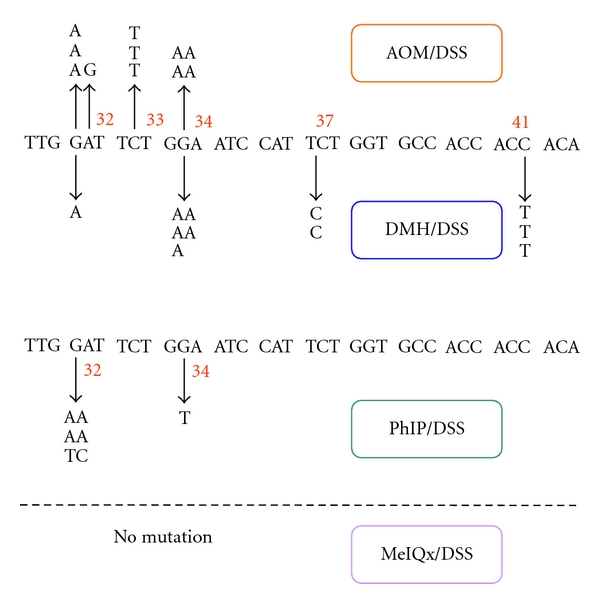
Mutations in the GSK-3*β* phosphorylation consensus motif of the *β*-*catenin* gene in adenocarcinomas of mice induced by AOM/DSS, DMH/DSS, PhIP/DSS, and 2-amino-3,8-dimethylimidazo-[4,5-*f*]-quinoxaline (MeIQx)/DSS. PhIP and MeIQx are heterocyclic amines.

Instead of AOM, experiments with DMH [23] or a heterocyclic amine, 2-amino-1-methyl-6-phenylimidazo[4,5-*b*]pyridine (PhIP) [24] as an initiator (colonic carcinogene) and followed by DSS treatment showed similar results described previously (Figure 13). Histopathologically, adenocarcinoma induced by DMH/DSS showed severer atypia and more aggressive biological natures than that induced by AOM/DSS. As noticed in the cancers induced by AOM/DSS, the adenocarcinoma cells developed in the inflamed colon of mice that received DMH and DSS were positive for COX-2, iNOS, and *β*-catenin (Figure 14). Mutation patterns of the **β*-catenin* gene were slightly among the adenocarcinomas that were induced by the different treatment regimens: AOM/DSS, codon 32–34, 37, and 41; DMH/DSS, codon 32, 34, 37, and 41; and PhIP/DSS, codon 32 and 34 (Figure 15). However, these mutations were restricted in the codon region (32–34, 37, 41, and 45) that played an important role in degradation of *β*-catenin protein. 

**Figure 16:**
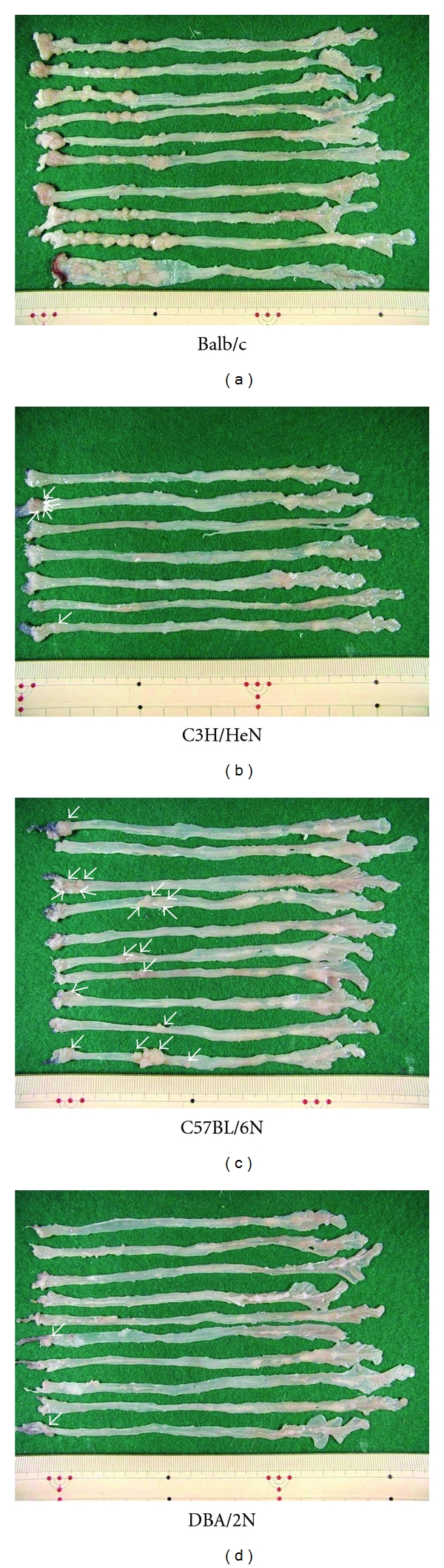
Macroscopic view of large bowel of four strains (Balb/c, C57BL/6N, C3H/HeN, and DBA/2N) of mice that received AOM and DSS.

There was a report of a difference in sensitivity of DSS-induced colitis among the species of mice [25]. To investigate whether the species differences influence inflammation-associated colorectal carcinogenesis, the sensitivity for different species of mice (Balb/c, C57BL/6N, C3H/HeN, and DBA/2N) were subjected to AOM/DSS-induced colorectal carcinogenesis [26]. The sensitivity to the AOM/DSS-induced colorectal carcinogenesis was as follows: Balb/c > C57BL/6N ≫ C3H/HeN = DBA/2N (Figure 16). The sensitivity was in relation to the nitrotyrosine-positive score estimated by immunohistochemical analysis, suggesting the importance of nitrotyrosine in the AOM/DSS-induced colorectal carcinogenesis [26]. 

**Figure 17:**
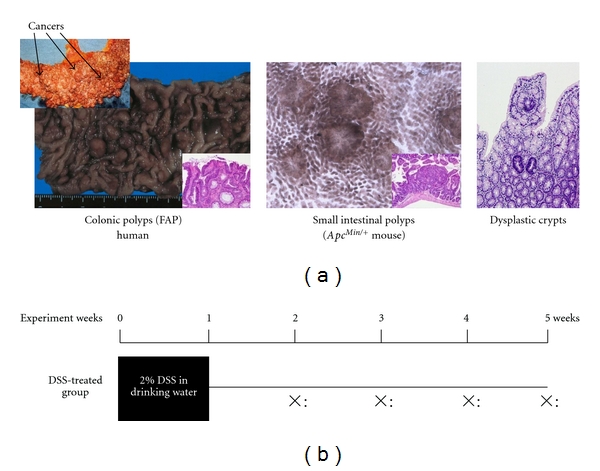
Colonic polyps in a familial adenomatous polyposis (FAP) patient and small intestinal polyps in an *APC^Min^* ^/+^ mouse (a). Experimental protocol for determining whether DSS promotes the growth of colonic dysplastic crypts in *APC^Min^* ^/+^ mice (b) [29].

**Figure 18:**
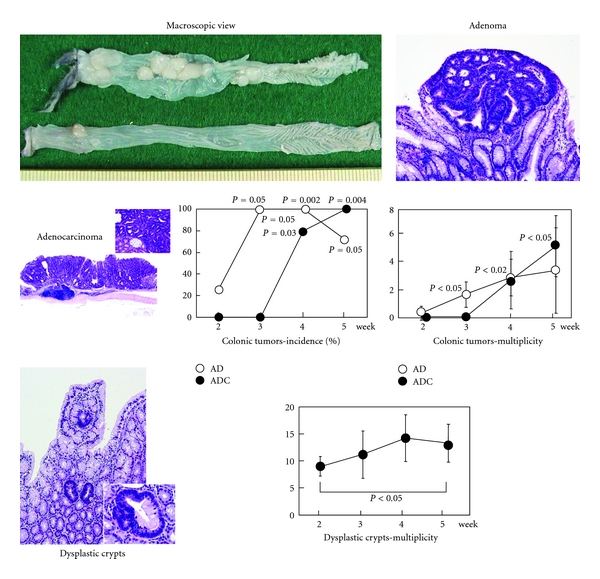
Macroscopic view and histopathology of colonic tumors and dysplastic crypts in *APC^Min^* ^/+^ mice that received 2% DSS for one week. Graphs show developments of these lesions during the study (up to 5 weeks).

**Figure 19:**
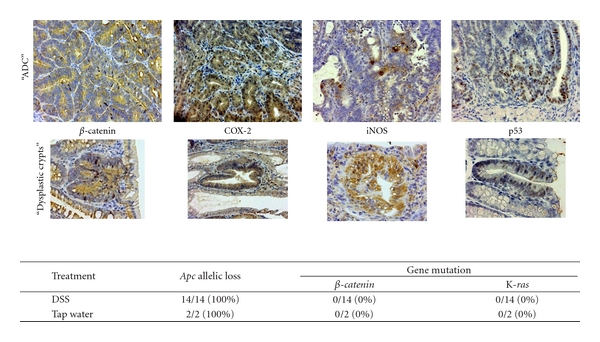
Immunohistochemistry of *β*-catenin, COX-2, iNOS, and p53 in the colonic adenocarcinoma and dysplastic crypts developed in male *Apc^Min^* ^/+^ mice that received 2% DSS (upper panel). *Apc* allelic loss and gene mutations of *β*-*catenin* and K-*ras* in the colonic adenocarcinoma from male *Apc^Min/+^* mice (lower panel).

In *Apc^Min/+^* mice, known as an animal model for familial adenomatous polyposis (FAP), multiple tumors (tubular adenomas) develop in the small intestine, instead of the large intestine in human FAP, and markedly few tumors develop in the large bowel. However, dysplastic crypts are observed in the colonic mucosa of *Apc^Min/+^* mice (Figure 17) [27, 28]. Therefore, DSS possibly enhances the growth of dysplastic crypts, and finally the lesions progress to adenocarcinomas. To investigate whether DSS-induced inflammation in the colonic mucosa would accelerate the growth of dysplastic crypts, *Apc^Min/+^* mice were given drinking water containing 2% DSS for one week without the initiation (carcinogen) treatment [29]. Surprisingly, multiple colorectal tumors, which were histopathologically tubular adenomas and adenocarcinomas, developed four weeks after the end of DSS treatment (Figure 18). Immunohistochemistry showed that the developed colorectal adenocarcinomas were positive against *β*-catenin, COX-2, iNOS, and p53 antibodies (Figure 19), suggesting that these factors were involved in the development of colorectal neoplasms in the *Apc^Min/+^* mice by the DSS treatment, in addition to oxidative stress and nitrosative stress. The findings suggested that DSS-induced inflammation in the large bowel of *Apc^Min/+^* mice exerts powerful tumor-promotion and/or progression effects on the growth of dysplastic crypts, which had already existed after the birth [27, 28].

Taken together, development of a mouse inflammation-associated colorectal carcinogenesis model was briefly described here, and the model was named as the TANAKA model. This model was possible to induce colorectal tumors in a short-term period in rats as well by similar treatment regimens (AOM/DSS and DMH/DSS) [30, 31]. It is anticipated that use of the TANAKA model will help advance the research on elucidation of the mechanisms of inflammation-associated colorectal carcinogenesis, inhibition of such carcinogenesis, and clarification of the mechanisms of the tumor-promotion ability of DSS. In particular, development of challenging research using Kyoto *Apc  *Delta (KAD) rats in Kyoto University will give new insight in the pathogenesis of colorectal cancer development in the inflamed coon [32].

## 4. Exploration of Chemopreventive Agents Using an Inflammation-Associated Colorectal Carcinogenic Model and Elucidation of the Mechanisms

Studies on chemoprevention of inflammation-associated colorectal carcinogenesis by several natural and synthetic compounds against have been reported using the AOM/DSS-induced mouse and rat colorectal carcinogenesis models. Several are promising compounds and their clinical application is expected. Representative compounds are auraptene and nobiletin from citrus fruits [33], collinin [33], *β*-cyclodextrin inclusion compounds of auraptene and 4′-geranyloxyferulic acid [34], tricin [35], melatonin [30], ursodeoxycholic acid [36], COX-2 selective inhibitor nimesulide [37], iNOS selective inhibitors [38], PPAR ligands (troglitazone and bezafibrate) [37], and a lipophilic statin pitavastatin [39]. All these compounds have anti-inflammatory activity and are able to suppress the expression of COX-2, iNOS, and inflammatory cytokines. 

## 5. Conclusions

Animal colorectal carcinogenesis models of our own making with the background of colitis mimicking human UC are introduced, and the exploration of chemopreventive compounds using these animal models is described. In addition, we confirmed upregulation of Wif1, Plat, Myc, and Plscr2 and downregulation of Pparbp, Tgfb3, and PPAR*γ* by comprehensive gene expression analysis in the colonic mucosa of mice that received AOM and DSS [40]. Moreover, proteomics analysis demonstrated that beta-tropomyosin, tropomyosin 1 alpha isoform b, and S100 calcium binding protein A9 were upregulated, while Car1, selenium-binding protein 1, HMG-CoA synthase, thioredoxin 1, 1 Cys peroxiredoxin protein 2, Fcgbp protein, Cytochrome c oxidase subunit Va, and ETHE1 protein were downregulated [41]. Significance of expression of these genes and proteins in inflammation-associated colorectal carcinogenesis remains poorly understood and further detailed analysis is required. Since our recent study demonstrated that NF-*κ*B and Nrf2 were expressed in not only inflammatory cells but also cancer cells in the TANAKA (AOM/DSS) model [34], these molecules may be the targets for cancer chemoprevention against colorectal cancer in the inflamed colon. Moreover, modification of the protocol of the TANAKA model may help us to detect environmental carcinogens [42] and tumor-promoters [43] for the large bowel. Fortunately, the animal models introduced here have attracted attention of young researchers that are doing research on colorectal carcinogenesis, IBD, inflammation, and cancer. It is anticipated that use of these models will advance elucidation of the mechanisms (methylation and microRNA) of inflammation-associated colorectal carcinogenesis, exploration of its suppression and mechanisms, and clarification of the mechanisms of tumor-promotion activity of DSS. 

## Figures and Tables

**Table 1:** Inflammation and cancer in various tissues.

Chronic inflammation	Site and associated cancer
Chewing tobacco, Oral irritation	Oral squamous cell carcinoma
Smoking, Chronic bronchitis, Chronic	Lung cancer
obstructive pulmonary disease	
Asbestosis	Mesothelioma
Reflux esophagitis, Barrett's esophagus	Esophageal adenocarcinoma
*H. pylori*-induced gastritis	Gastric cancer, Mucosa-associated lymphoid tissue lymphoma
Chronic pancreatitis	Pancreatic adenocarcinoma
Viral (Hepatitis B and C virus) hepatitis	Hepatocellular carcinoma
Opisthorchis sinensis infection (liver fluke)	Cholangio carcinoma
Inflammatory bowel disease (IBD)	Colorectal adenocarcinoma
Pelvic inflammatory disease	Ovarian cancer
Human papilloma virus (HPV) infection	Anogenital carcinoma
Schistosomiasis	Bladder cancer
Chronic scar tissue	Scar cancer arising in pre-existing scars in the lung, skin, and other tissues
Human herpes simplex virus type 8	Kaposi sarcoma
Chronic oesteomyelitis	Osteosarcoma

**Table 2:** Animal models of colorectal carcinogenesis and inflammatory bowel disease. HCAs: heterocyclic amines.

(1) Animal models of colorectal carcinogenesis	
(i) *Carcinogen-induced animal models *	
Azoxymethane (AOM)	
1,2-Dimethyl-hydrazine (DMH)	
HCAs: 2-Amino-1-methyl-6-phenylimidazo[4,5-*b*]pyridine (PhIP)	
2-Amino-3,8-dimethylimidazo[4,5-*f*] quinoxaline (MeIQx)	
(ii) *Mutant, transgenic, knockout animal models *	
Min mouse and APC^Δ474^ knockout mouse	

(2) Animal models of inflammatory bowel disease	

(i) *Chemically and polymer-induced models *	
Trinitrobenzene sulfonic acid (TNBS): rat, mouse, rabbit	
Dextran sulfate sodium (DSS): rat, mouse, hamster	
Carrageenan: mouse, guinea pig, rabbit	
(ii)* Microbial-induced models *	
Cotton-top tamarins (*Saguinus oedipus*)	
(iii) *Mutant mice *	
IL-2^−/−^, IL-10^−/−^, TCR-*α* ^−/−^, TCR-*β* ^−/−^	
